# Impact of discontinuation of cephazolin prophylaxis on the incidence of postoperative adverse events in cataract surgery

**DOI:** 10.1186/s40780-025-00412-x

**Published:** 2025-01-30

**Authors:** Kazutaka Uchida, Keita Takahama, Kenshiro Higashi, Yuki Kusano, Koki Matsumoto, Yoshihiro Nakagawa, Kazutaka Oda

**Affiliations:** 1 Department of Pharmacy, Kumamoto Shinto General Hospital, 3-2-65, Oe, Chuo-ku, Kumamoto city, Kumamoto, 862-8655 Japan; 2 Department of Ophthalmology, Kumamoto Shinto General Hospital, 3-2-65, Oe, Chuo-ku, Kumamoto city, Kumamoto, 862-8655 Japan; 3 Department of Pharmacy, Kumamoto University Hospital, 1-1-1, Honjo, Chuo-ku, Kumamoto city, Kumamoto, 860-8556 Japan

**Keywords:** Cataract surgery, Cefazolin, Surgical site infections, Adverse events

## Abstract

**Background:**

Cataract surgeries are increasing annually, making appropriate medical management essential. The routine use of systemic antimicrobial agents for preventing surgical site infections lacks strong evidence and may increase the risk of drug-resistant bacteria and adverse events. This study examined the impact of discontinuing cefazolin (CEZ) administration during the perioperative period of cataract surgery on the incidence of postoperative adverse events and medical costs.

**Methods:**

Inpatient cataract surgery patients were divided into two groups: the CEZ-use group (April 2021 to March 2022) and the non-CEZ-use group (April 2022 to March 2023). The primary endpoints were the incidence of adverse events and medical costs, while the secondary endpoint was the incidence of endophthalmitis.

**Results:**

A total of 265 patients were in the CEZ group, and 316 were in the non-CEZ group. Six postoperative adverse events (2.3%, 95% confidence interval: 0.8–4.9) occurred in the CEZ group, with an estimated 230 patients (80–490 from the 95% confidence interval) expected to experience adverse events per 10,000 patients using CEZ. The non-CEZ group had no adverse events and reduced drug costs by approximately 46,000 yen. Insurance claim amounts were also reduced. No cases of early postoperative endophthalmitis were observed in either group.

**Conclusions:**

Discontinuation of CEZ prophylaxis during the perioperative period of cataract surgery effectively reduced the risk of adverse events. Medical for the period after discontinuing CEZ did not increase.

**Trial registration:**

Retrospectively registered.

## Background

The number of cataract surgeries is increasing annually, with approximately 1 million procedures performed each year. As the population ages, this number is expected to rise, underscoring the importance of appropriate medical management.

Postoperative endophthalmitis is one of the most significant surgical site infections (SSI) in cataract surgery. Although rare, it can lead to poor visual outcomes. Evidence shows that povidone-iodine disinfection during surgery effectively reduces bacterial contamination of the anterior chamber [1], a practice widely adopted by many institutions. The primary causative organisms of postoperative endophthalmitis include coagulase-negative *Staphylococci*, *Staphylococcus aureus*, *Streptococcus* spp., *Enterococcus faecalis*, and *Propionibacterium* spp [2, 3]. Various antimicrobial agents have been studied to prevent infections caused by these organisms. However, the use of antimicrobial agents must be carefully considered due to the risks of adverse events such as anaphylaxis, allergic reactions, and diarrhea.

Recently, numerous international reports have emphasized the effectiveness of intravitreal cefuroxime in the anterior chamber, while evidence supporting systemic administration remains insufficient [4]. In Japan, studies on prophylactic antimicrobial use during cataract surgery have shown promising results. Matsuo et al. demonstrated that properly administered topical antimicrobial agents, without systemic prophylaxis, successfully prevented early postoperative endophthalmitis, although the sample size was limited, leaving room for further research [5]. Likewise, Takahashi et al. reported no cases of early endophthalmitis following the discontinuation of postoperative oral antimicrobial agents [6].

The unnecessary use of antimicrobial agents can contribute to the rise of drug-resistant bacteria. Japan’s Antimicrobial Resistance Action Plan, published in 2016 [7], advocates for the reduction and appropriate use of antimicrobial agents, with a new plan introduced in 2023 [8]. In the context of cataract surgery, unnecessary antimicrobial use during the perioperative period may cause allergic reactions, such as anaphylaxis, adverse events like diarrhea, and promote drug-resistant bacteria—risks that outweigh the benefits of SSI prevention. At Kumamoto Shinto General Hospital, the Department of Ophthalmology included cefazolin (CEZ) in its clinical pathway (CP) for perioperative cataract surgery until March 2022, administering a single dose during the perioperative period. This practice was discontinued in April 2022 following consultation with an ophthalmologist. However, there are few reports assessing the impact of discontinuing CEZ administration on patient safety and medical and drug costs in the perioperative cataract CP. While the increase in adverse events in the CEZ group is a well-established fact, we have further focused on detailing the specific adverse events related to cefazolin administration, particularly their frequency and associated medical and drug costs, in the context of perioperative cataract CP.

This study aims to evaluate the impact of discontinuing CEZ use during the perioperative period of cataract surgery on the incidence of postoperative adverse events and medical costs.

## Patients and methods

### Study design, participants, and data collection

This retrospective cohort study was conducted at Kumamoto Shinto General Hospital using medical records. Ethical guidelines in accordance with the *Ethical Guidelines for Medical Research Involving Human Subjects* were followed, and approval was obtained from the Ethics Committee of Kumamoto Shinto General Hospital (Approval number: 2023-J01-002).

Patients were classified into two groups: those who received CEZ from April 1, 2021, to March 31, 2022 (CEZ group), and those who did not receive CEZ from April 1, 2022, to March 31, 2023 (non-CEZ group). Patients who underwent concurrent cataract surgery and corneal transplantation were excluded. The antibacterial eye drops—Levofloxacin 1.5%— were used for 3 days before surgery. CEZ was administered via a one-hour infusion. The primary endpoints were the incidence of postoperative adverse events and medical costs; the secondary endpoint was the occurrence of SSI. Adverse events in this study were defined as cases where medical records documented allergic or gastrointestinal symptoms such as anaphylaxis, skin rash, or other allergic reactions occurring between surgery and discharge. This approach was applied consistently for all patients in both groups. Patients who used laxatives were excluded from the analysis criteria for abdominal pain and diarrhea. SSI was defined as significant anterior chamber inflammation (e.g., anterior chamber abscess, fibrin precipitation) within one week post-surgery [9, 10], with a physician’s diagnosis of early-onset endophthalmitis after cataract surgery. Medical costs were calculated based on fiscal year 2021 prices of CEZ and insurance claim amounts during hospitalization, stratified by bilateral eyes and unilateral eye surgeries. Insurance claim amounts included basic hospitalization charges, surgical charges, meal charges, private room charges, and various instructional charges. The length of stay for hospitalization was also assessed.

The occurrence of endophthalmitis was monitored by an attending doctor at 7–10 days post-discharge. SSI was confirmed through clinical signs of anterior chamber inflammation within one week of surgery and a physician’s diagnosis of early postoperative endophthalmitis. Data were collected on patient age, gender, history of allergies, body mass index (BMI), surgical site during hospitalization, and medical history, including diabetes, dialysis, and use of steroids, immunosuppressive drugs, or anticancer drugs. Additional data included preoperative eye examination results, length of stay for hospitalization, duration of postoperative antibacterial eye drop use, and the number of CEZ doses administered during cataract surgery.

### Statistical analysis

Patient demographics were compared using Welch’s t-test for continuous variables with a normal distribution and the Mann-Whitney U test for non-normally distributed continuous variables. Fisher’s exact test was applied to categorical variables. The composite incidence rate of postoperative adverse events was estimated with confidence intervals. A *p*-value of less than 0.05 was considered statistically significant. All statistical analyses were performed using EZR version 2.9-1.

## Results

### Patient background, incidence of early postoperative endophthalmitis, antimicrobial use, and medical costs

The baseline characteristics of the patients are shown in Table 1. A total of 265 patients underwent cataract surgery in the CEZ group, while 316 patients were in the non-CEZ group. There were no statistically significant differences between the groups in terms of gender, age, history of allergies, number of operated eyes, history of diabetes, dialysis, or use of immunosuppressive medications. However, the mean (standard deviation) BMI was 23.8 (4.0) kg/m² in the CEZ group and 23.1 (3.3) kg/m² in the non-CEZ group, with a statistically significant difference (*P* = 0.04). We confirmed that hand washing and disinfection methods remained consistent between the two groups.

**Table 1:** Demographics of the participants

	CEZ group *n* = 265	Non-CEZ group *n* = 316	*P* value
Age, years	76 (8.9)	77 (8.2)	0.06
Male/female, (male%)	101/164 (38.1%)	120/196 (38%)	1.00
Body mass index, kg/m^2^	23.8 (4.0)	23.1 (3.3)	0.04
Number of operated eyes			
Unilateral eye/bilateral eyes, (unilateral eye%)	105/160 (39.6%)	125/191 (39.6%)	1.00
History of allergies			
Antibiotics, n (%) Non-antibiotics, n (%) Foods, n (%)	5 (1.9%) 16 (6.0%) 18 (6.8%)	10 (3.2%) 28 (8.9%) 19 (6.0%)	0.43 0.21 0.74
Diabetes mellitus, n (%)	60 (22.6%)	83 (26.3%)	0.33
HbA1c, %	6.8 (1.0)	6.8 (0.8)	0.81
Hemodialysis, n (%)	4 (1.5%)	3 (0.9%)	0.71
Concurrent medication			
Steroids, n (%)	16 (6.0%)	18 (5.7%)	0.86
Immunosuppressants, n (%)	10 (3.8%)	12 (3.8%)	1.00
Anticancer drugs, n (%)	7 (2.6%)	6 (1.9%)	0.58
Patients with preoperative eye drops, patient (%)	264 (99.6%)	311 (98.4%)	0.23
Length of stay for hospitalization			
Unilateral eye surgery	3 [2–8]	2 [2–6]	< 0.01
Bilateral eyes surgery	6 [4–9]	5 [2–12]	< 0.01
Duration of postoperative antibiotic eye drops, months	1 [1–3]	1 [1–4]	< 0.01

Data were depicted in mean (standard deviation) or median [min–max]

The median (min–max) length of stay for hospitalization for unilateral eye surgery was 3 (2–8) days in the CEZ group and 2 (2–6) days in the non-CEZ group (*P* < 0.01). For bilateral eyes, the median hospital stay was 6 (4–9) days in the CEZ group and 5 (2–12) days in the non-CEZ group (*P* < 0.01). The median (min–max) duration of postoperative antimicrobial eye drop use was 1 (1–3) months in the CEZ group and 1 (1–4) months in the non-CEZ group, with a statistically significant difference (*P* < 0.01).

### Postoperative adverse events and insurance claim amount

Table 2 represents the results. During the perioperative period, no adverse events were reported in the non-CEZ group. However, six adverse events were recorded in the CEZ group. These included three cases of diarrhea, two cases of nausea, and one case of skin rash. The overall postoperative adverse event rate in the CEZ group was 2.3% (6 out of 265 patients, 95% confidence interval: 0.8–4.9). We observed that, in the CEZ group, 3 patients developed diarrhea without the use of laxatives. Among patients who did use laxatives, diarrhea was observed in 10 (13.7%) of the CEZ group and 8 (10.8%) of the non-CEZ group. No cases of early postoperative endophthalmitis were observed in either group during the study period.

**Table 2:** Postoperative adverse events and insurance claim amount

	CEZ group *n* = 265	Non-CEZ group *n* = 316	*P* value
Postoperative adverse event, n	6 (2.3%, 95% confidence interval: 0.8–4.9)	0	N.C.
Insurance claim amount, yen			
Unilateral eye surgery Bilateral eyes surgery	234,298 (38,756) 434,887 (26,340)	211,322 (33,485) 411,887 (48,532)	< 0.01 < 0.01

Data were depicted in mean (standard deviation). *N.C.*, not calculated

The total amount of CEZ used during the perioperative period for cataract surgery decreased from 265 g to 0 g after its discontinuation, resulting in a cost reduction of approximately 46,000 yen. For bilateral eyes surgery, the mean (standard deviation) insurance claim amount were 434,887 (26,340) yen in the CEZ group, compared to 411,887 (48,532) yen in the non-CEZ group (a mean reduction of 23,000 yen, *P* < 0.01). For unilateral eye surgery, these figures were 234,298 (38,756) yen in the CEZ group, compared to 211,322 (33,485) yen in the non-CEZ group (a mean reduction of 22,976, *P* < 0.01).

## Discussion

This study observed no cases of SSI, either during CEZ administration or after its discontinuation. A previous study reported a 0.0245% incidence of early postoperative endophthalmitis, with a 95% confidence interval of 0.0113–0.0377% [11]. This translates to an estimated 2.45 cases per 10,000 patients (with a 95% confidence interval of 1.13–3.77 cases per 10,000). It is understandable that SSI was not observed in the relatively small sample size of our study. Consequently, this finding cannot be attributed to the discontinuation of CEZ. To further explore this topic, a larger-scale study would be required to comprehensively evaluate the relationship between CEZ discontinuation and the incidence of SSI. Importantly, because domestic guidelines recommend intravenous administration of CEZ for cataract surgery in patients with risk factors, we acknowledged that a cautious approach may be advisable for such patients.

Meanwhile, the postoperative adverse event rate in the CEZ group was 2.3% (95% CI: 0.8–4.9%). This suggests that 80–490 adverse events per 10,000 patients could potentially be avoided if CEZ was not used during the perioperative period. Takahashi et al. reported that discontinuing postoperative oral antibiotics in cataract surgery reduced perioperative adverse events [6]. Similarly, Elliman et al. highlighted that prophylactic antibiotics increased the risk of acute kidney injury and *Clostridioides difficile* infection, depending on the timing of administration [12]. One serious adverse event of CEZ is anaphylaxis, a rare but potentially life-threatening condition that requires immediate intervention. Cephem antibiotics, including CEZ, are known to cause anaphylaxis more frequently than other antimicrobial agents [13, 14]. Takazawa et al. reported that perioperative anaphylaxis occurred in approximately 1 in 10,000 cases [15], while the Japanese Society of Anesthesiologists found the incidence to be 1 in 40,000 cases. In France, antimicrobial agents were implicated in 12.8% of anaphylaxis cases during general anesthesia [16]. Although no cases of anaphylaxis were recorded in this study, caution is warranted when administering antibiotics. Regarding the definition of diarrhea excluding patients who used laxatives, while it is not possible to entirely rule out the influence of CEZ administration on diarrhea in patients who used laxatives, laxatives represent a significant confounding factor, introducing bias in evaluating diarrhea related to CEZ. For this reason, we believe the exclusion of patients using laxatives was an appropriate methodological decision for this study.

The discontinuation of CEZ in the perioperative period for cataract surgery may reduce the burden on medical staff, lower the medical costs associated with antibiotic-related adverse events, and eliminate the need for intravenous lines before surgery, which in turn could reduce the risk of neurological injury and infection. However, there are no reports evaluating the economic outcomes of perioperative antibiotic use in cataract surgery, such as increased costs or extended hospital stays due to adverse events. Our study demonstrated that the discontinuation of CEZ did not increase insurance claim amounts. Instead, claim amounts were significantly reduced. While the direct relationship between the discontinuation of CEZ and the reduction in claim amounts was minimal, the reduction can be primarily attributed to shorter length of stay for hospitalization. Further research is warranted to explore this relationship in greater detail.

There are several limitations to this study. Because this was a retrospective study, it was not possible to establish a definitive causal relationship between CEZ use and postoperative adverse events. Moreover, we could not assess adverse events such as abnormal laboratory findings since no postoperative blood tests were performed. Lastly, the retrospective nature of this study may have limited the identification of all possible adverse events.

## Conclusions

We reported that discontinuation of CEZ in the perioperative period of cataract surgery effectively reduces the risk of postoperative adverse events without any observed cases of SSI. Medical costs for the period after discontinuing CEZ did not increase.

## Data Availability

Data and materials related to this study are available from the corresponding author upon reasonable request.

## Abbreviations

BMI: Body mass index
CEZ: Cefazolin
CP: Clinical pathway
SSI: Surgical site infections
